# Single versus Double Coffee-Ring Effect Patterns in Thin-Layer Chromatography Coupled with Surface-Enhanced Raman Spectroscopic Analysis of Anti-Diabetic Drugs Adulterated in Herbal Products

**DOI:** 10.3390/molecules28145492

**Published:** 2023-07-18

**Authors:** Dao Thi Cam Minh, Le Thi Bao Tram, Nguyen Hai Phong, Hoang Thi Lan Huong, Le Van Vu, Le Anh Thi, Nguyen Thi Kieu Anh, Pham Thi Thanh Ha

**Affiliations:** 1 Faculty of Pharmacy, University of Medicine and Pharmacy, Hue University, Hue 530000, Vietnam; dtcminh@hueuni.edu.vn (D.T.C.M.); ltbtram@hueuni.edu.vn (L.T.B.T.); 2 Department of Chemistry, University of Sciences, Hue University, Hue 530000, Vietnam; nhphong@hueuni.edu.vn; 3 Drug, Cosmetic and Food Quality Control Center of Thua Thien Hue Province, Hue 530000, Vietnam; lanhuong.kiemnghiem@gmail.com; 4 Faculty of Physics, VNU University of Science, Hanoi 100000, Vietnam; levanvu@hus.edu.vn; 5 Institute of Research and Development, Duy Tan University, Danang 550000, Vietnam; 6 Faculty of Natural Sciences, Duy Tan University, Da Nang 550000, Vietnam; 7 Department of Analytical Chemistry and Drug Quality Control, Hanoi University of Pharmacy, Hanoi 100000, Vietnam; anhntk@hup.edu.vn

**Keywords:** anti-diabetic drug, TLC-SERS, coffee ring effect, silver nanoparticles, adulteration, herbal products

## Abstract

In thin-layer chromatography coupled with surface-enhanced Raman spectroscopy (TLC-SERS), the coffee ring effect (CRE) describes the formation of a ring-shape spot (blank in the middle and darker on the edge) caused by the aggregation of silver nanoparticles (Ag NPs), alone (single CRE) or with the analytes (double CRE). In this work, the SCRE and DCRE were investigated in two anti-diabetic drugs, hydrophobic glibenclamide (GLB) and more hydrophilic metformin (MET). The SCRE occurred in GLB analysis, as opposed to the DCRE that occurred in MET. It was proven that for optimization of the TLC-SERS analytical procedure, it is necessary to distinguish the CRE patterns of analytes. Additionally, MET and GLB were analyzed with the developed TLC-SERS method and confirmed by another validated method using high-performance liquid chromatography. Four herbal products collected on the market were found to be adulterated with GLB or/and MET; among those, one product was adulterated with both MET and GLB, and two products were adulterated with GLB at a higher concentration than the usual GLB prescription dose. The TLC-SERS method provided a useful tool for the simultaneous detection of adulterated anti-diabetic herbal products, and the comparison of the SCRE and DCRE provided more evidence to predict CRE patterns in TLC-SERS.

## 1. Introduction

For decades, adulteration of synthetic drugs in herbal products has been an issue of concern. It is a common belief that herbal products cause fewer adverse reactions compared to synthetic drugs when used for a long time. Therefore, for chronic diseases such as diabetes, the Asian community prefers using traditional remedies and herbal products. However, falsified herbal products have been found on the market, resulting in serious hazards to public health due to unexpected adverse reactions caused by unknown abuse of synthetic drugs. For an analytical method with the purpose of adulteration detection, selectivity is the most important criterion. Hyphenated techniques are commonly used, typically chromatography (liquid, gas, or thin-layer chromatography - LC, GC, TLC) coupled with Ultraviolet-visible (UV–Vis), infrared (IR), Raman scattering (RS), or mass spectroscopy (MS) [1]. However, for confirmatory detection of adulteration, the spectroscopic technique should be of high specificities, such as IR, RS, or MS. Therefore, gas chromatography coupled to mass spectroscopy (GC-MS), liquid chromatography coupled with tandem mass spectroscopy (LC-MS/MS), or ultra-performance liquid chromatography coupled with time of flight (UPLC-TOF) have been preferable techniques, and more recently thin-plate chromatography and surface-enhanced Raman scattering (TLC-SERS) was also suggested as a selective detection method for confirmatory results [1]. TLC-SERS methods have been developed recently to detect a variety of synthetic drugs adulterated in herbal products among other applications [2,3,4]. Especially for the application in the detection of chemical compounds adulterated in herbal products, TLC-SERS showed significant advantages of the hyphenated technique. In many cases, the fluorescence of herbal compositions can be so high that the signal is over-ranged and the Raman spectrum is masked [5]. The TLC separation minimizes the fluorescence of complex herbal compositions, enabling the SERS measurement of the separated analyte of interest. On the other hand, the high specificity of the Raman spectrum and the high sensitivity of SERS detection compensates very well for the selectivity and sensitivity of TLC methods. 

Although TLC-SERS has promising potential for fast analysis in a complex system, the sensitivity, reproducibility, and stability of this technique still need to be developed. To obtain high Raman enhancement, after the TLC stage and before the SERS stage, the colloidal silver nanoparticle (AgNP) solution is dripped on the active spot on the plate, and the laser beam should be located at the “hotspot” with the highest aggregation of both analyte particles and AgNPs. This colloidal AgNP instillation produces a coffee ring effect (CRE)—a formation of a ring-shaped spot, similar to a coffee stain (blank in the center, and darker on the edge), as reported in previous studies [3,6]. Typically, CRE-driven particle dethronement commences once the radial flow begins bringing the droplet solution to the bound of the three-phase contact line and at the same time taking colloidal particles away from the center area, resulting in their accumulation at the forming ring stain [3,7]. The TLC-SERS method with the CRE has been advanced for the quantitative analysis of complex systems and is widely considered an instrument for the self-assembly of nanostructures, including colloidal metal particle suspension and immobilization on solid substrates [8,9,10]. Increasing evidence showed that the assembled metal nanoparticles in the area of a coffee ring form numerous “hotspots”, leading to the enhancement of the Raman signal, and the formed CRE ensures both detection sensitivity and reproducibility [11,12]. The CRE ring stain may result from either AgNPs alone (single coffee ring effect—SCRE) [13], or both AgNPs and analyte (double coffee ring effect—DCRE) [14]. In this study, the occurrence of CRE patterns (SCRE and DCRE) in TLC-SERS methods for the detection of adulterants in herbal products was analyzed for two compounds with blood glucose-lowering effects: glibenclamide (GLB), as a hydrophobic compound, and metformin (MET), as a more hydrophilic compound. Both compounds are the most commonly used anti-diabetic drugs and can be potential adulterants in herbal products for blood sugar-lowering treatment. To examine the hydrophobicity–CRE pattern relevance, the current study was designed to fulfill the following aims: (1) determine the CRE pattern of GLB and MET in TLC-SERS performance; (2) optimize the TLC-SERS analytical procedure according to the CRE pattern determined; and (3) apply the developed TLC-SERS method for analysis of GLB and adulterated MET herbal products examined in parallel with a validated high-performance liquid chromatographic (HPLC) method.

## 2. Results

### 2.1. Preliminary TLC-SERS Method Development

#### 2.1.1. Thin Layer Chromatographic Design 

**Figure 1:**
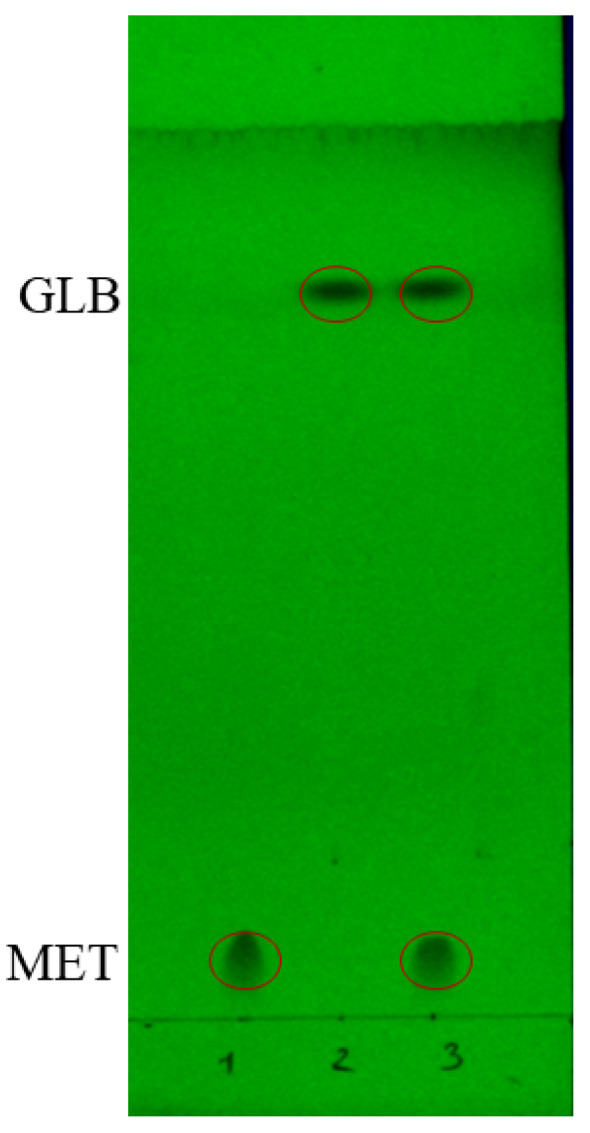
Chromatogram obtained at 254 nm detection of (1) MET standard solution; (2) GLB standard solution; (3) MET and GLB mixture standard solution.

MET and GLB stock standard solutions at 1 mg/mL in methanol were prepared separately. TLC analyses were conducted on a silica gel 60 F254 TLC plate, with a sample volume of 5 µL, and the isolated spots were visualized under ultraviolet illumination at 254 nm. The n-butyl acetate/methanol/formic acid (11:2.5:1.5) was used as a mobile phase solvent; the two analytes were reciprocally separated, and obtained analyte spots were marked as shown in Figure 1. The R_f_ values of MET and GLB on the TLC plate were 0.05 and 0.77, respectively. The values of R_f_ were distinguished clearly, which might be advantageous for SERS detection, and this mobile phase was used in the subsequent analysis.

#### 2.1.2. Silver Colloid Solution Preparation

The colloidal silver suspension was prepared according to previously published research [11]: 94 mL of distilled water was heated to 100 °C under a reflux condenser with vigorous magnetic stirring. After boiling, 2 mL of 0.1 M silver nitrate solution was dropwise added, followed by 4 mL of 1% sodium citrate solution. Reflux heating was maintained for 40 min then naturally cooled down to room temperature to obtain nano-silver colloid. The silver colloid was stored in the refrigerator and used for 6 months. 

**Figure 2:**
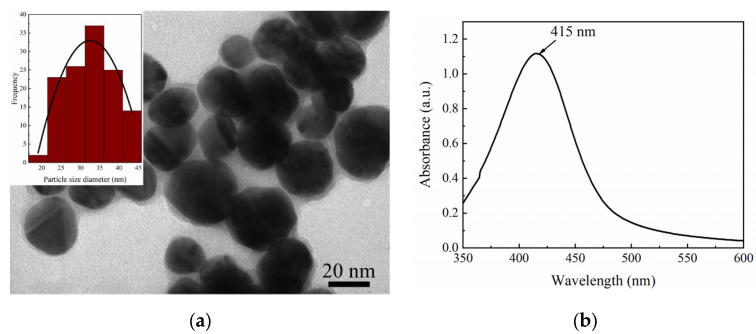
(**a**) TEM image of silver colloid for characterization of AgNP size, (**b**) UV–Vis spectrum of AgNPs.

Figure 2 presents (a) the transmission electron microscopy (TEM) and (b) UV–Vis spectroscopy of the silver colloid solution. In Figure 2a, the TEM image illustrates the morphology of the silver nanoparticles (AgNPs), with sizes ranging from 25 to 40 nm and an average size of 35 nm. The UV–Vis absorbance peak was detected at 415 nm as shown in Figure 2b, and the full width at half maximum (FWHM) of approximately 76 nm shows a relatively good uniformity of AgNP size. Both size and uniformity of AgNPs indicated good characteristics for the formation of “hotspots” [15,16]. These characteristics were maintained relatively stable for 6 months, which represented acquired stability for TLC-SERS measurement.

#### 2.1.3. Selection of Typical Raman Shifts for SERS Analysis

**Figure 3:**
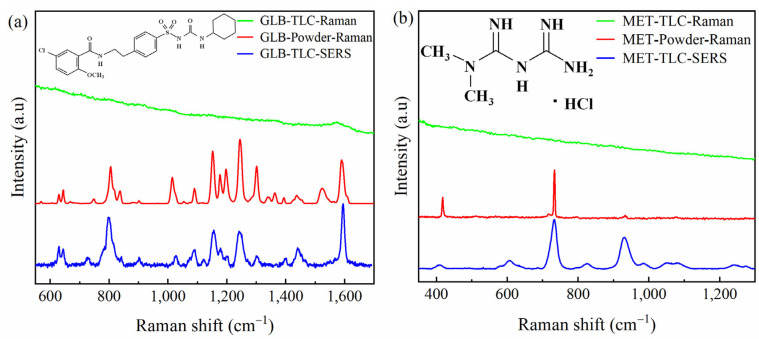
Normalized Raman and SERS spectra of (**a**) GLB and (**b**) MET, in which the red lines present the Raman spectra of standards in powder, the green lines present the Raman spectra of the standards on the TLC spot without AgNP enhancement, and the blue lines present the SERS spectra of the standards on the TLC spot after adding AgNPs for enhancement.

For the identification of typical Raman shifts for MET and GLB, the direct Raman spectra were recorded on powders of MET and GLB reference standards. The normalized Raman spectra of powders are presented as the red line in Figure 3, (a) for GLB and (b) for MET.

To verify the enhancement effect of the silver colloid, after TLC separation (as presented in Section 2.1.1), the Raman spectrum of each marked spot on the TLC plate was measured directly without adding silver colloid. Although the standard solutions were at a visible level under UV light, the Raman shifts were not visible for both MET and GLB. The obtained signals were the same as the signals of the silica base (green lines in Figure 3). The aqueous colloidal silver suspension (1.5 μL) prepared (as presented in Section 2.1.2) was dripped directly to the marked spots, and SERS signals were measured, with an excitation wavelength of 633 nm and a recording time of 20 s for MET and 60 s for GLB. The normalized SERS spectra of GLB and MET are shown in Figure 3 with blue lines.

Comparing the SERS and Raman spectra, it was observed that the typical shifts of both GLB and MET in SERS spectra were in good compliance with those of Raman spectra. For MET, the SERS spectra provide even more peaks for identification. The assignments of the vibration mode are listed in Table 1. All peaks observed in Raman spectra measured in the current study were in agreement with those reported in the literature [17,18,19].

For GLB, when comparing SERS and Raman spectra (Figure 2a, among 15 peaks in the range of 500 to 1700 cm^−1^, three peaks were the same (difference of 0 to 1 cm^−1^), six peaks had a minor difference of 2 to 4 cm^−1^, three peaks had a difference of 5 to 9 cm^−1^, and only two peaks had a difference of over 10 cm^−1^ between two spectra (Table 1). The main SERS peaks were assigned to C-Cl stretching, C-H bending, C-O stretching, sulfonyl stretching, C=C stretching, and C=O stretching at 643, 802, 1025, 1155, 1441, and 1592 cm^−1^, in agreement with other reports as shown in Table 1. The enhancement with SERS measurement was observed with improved intensities of some peaks such as 643, 802, 1441, and 1592 cm^−1^ (Figure 2a. Among 15 peaks recorded on the SERS spectrum of GLB, five peaks were selected as typical Raman shifts for GLB detection in TLC-SERS analysis. These peaks possess high intensity, robust frequency, and minor differences compared to the original Raman spectrum: 643; 802; 1155; 1241, and 1593 cm^−1^.

In the case of MET, peaks of Raman shifts in the SERS spectrum were in good accordance with the Raman spectrum (Table 1): two peaks were at the same shifts (difference of 0 to 1 (cm^−1^)), and one peak at low frequency with a difference of 9 cm^−1^. As shown in Figure 2b, the enhancement was well illustrated with higher intensities of peaks at 606, 826, 987, 1047, 1081, 1240, and 1275 cm^−1^ in the SERS spectrum compared to the Raman spectrum. The peaks are assigned to C-N-C deformation below 700 cm^−1^, while some peaks are in the range of 700–1000 cm^−1^ ascribable N-H wagging. The C-N stretching can be for 1000–1300 cm^−1^ region. For MET, three peaks with high intensity and robust signals were chosen for typical Raman shifts: 606; 733, and 932 cm^−1^.

### 2.2. Determination of CRE Pattern

Since the silver colloid suitable for anti-diabetic drugs was an aqueous solution, the solubility in the silver colloid solution would be low for GLB and higher for MET. As a result, we hypothesized that GLB distribution after TLC development would not be influenced by the addition of silver colloid, which means the SCRE was expected for GLB. In the meantime, the addition of aqueous silver colloid might partially dissolve the MET particles on the TLC spots, and redistribution of MET would occur, which means DCRE was expected for MET.

The stock solutions of GLB and MET (1 mg/mL) were used in the determination of CRE patterns. After TLC development, the GLB and MET spots were detected using 254 nm UV illumination. The CRE can be observed after a drop of AgNP solution. Since AgNPs were visible under normal sunlight, To determine the CRE pattern of each analyte during TLC-SERS analysis, after dripping nano-silver colloid onto the marked TLC spot, the distribution of AgNPs was observed under natural daylight, and the redistribution of analyte particles was observed under 254 nm UV light. The AgNP assembly in a narrow area of the CRE led to ”hotspot” formation, which would be propitious for SERS enhancement. Thus, the SERS performance was measured at different positions along the diameter of the coffee ring formed by silver colloids, and its relative distance to the TLC spot was studied to optimize the CRE-TLC-SERS method.

#### 2.2.1. CRE Pattern of Glibenclamide

**Figure 4:**
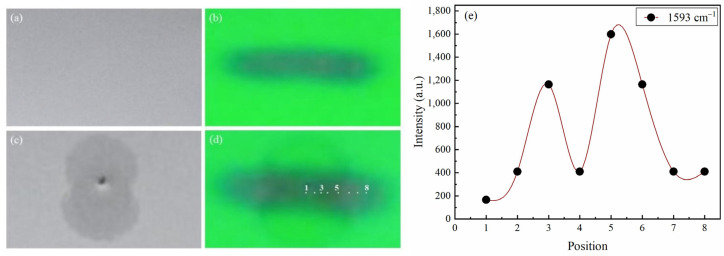
SCRE effect when applying silver colloid at the center of GLB stain: Image of GLB stain on TLC under normal light before (**a**) and after applying silver colloid (**c**); under 254 nm light before (**b**) and after applying silver colloid (**d**); SERS intensity of GLB at 1593 cm^−1^ the peak at different laser positions (**e**).

**Figure 5:**
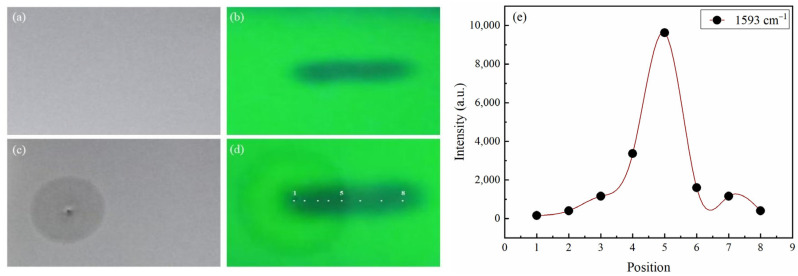
SCRE effect when applying silver colloid at the edge of GLB stain: Image of GLB stain on TLC under normal light before (**a**) and after applying silver colloid (**b**); under 254 nm light before (**c**) and after applying silver colloid (**d**) at the site of the stain; SERS intensity of GLB at 1593 cm^−1^ peak at different laser positions (**e**).

The CRE pattern of GLB was illustrated in Figure 4 and Figure 5, where (a) and (c) represent photos of the TLC plate under normal sunlight to observe AgNPs, and (b) and (d) were photos of TLC plate under 254 nm to observe GLB particles. Coffee rings of AgNPs were captured in Figure 4c and Figure 5c. The difference between Figure 4 and Figure 5 was the position of the silver colloid introduced at the center (Figure 4) or on the edge (Figure 5) of the GLB spot.

GLB stain was pictured as the long black pot in Figure 5b,d under 254 nm light. Comparing the black spots in Figure 4d and Figure 5d to those in Figure 4b and Figure 5b, it was shown that GLB stains remained in the same Gaussian distribution before and after adding silver colloids (Figure 5). This meant GLB was hardly redistributed under silver colloid droplets, or in other words, the SCRE occurred with GLB, as predicted.

Another interesting observation should be noticed in Figure 4. When colloidal silver is applied at the center of the GLB spot, the AgNP stain was a coffee-stain pattern, but not exactly a circular “ring shape”, but rather a fat “peanut shape” as shown clearly in Figure 4c and slightly on Figure 4d. This phenomenon further confirmed the SCRE pattern for GLB. Because GLB is hydrophobic, GLB particles were not soluble in aqueous silver colloid, creating a hindrance for Ag NPs movement on the TLC plate. Moreover, the GLB spot after TLC development was in typical Gaussian distribution, which meant the density of GLB particles and the center of the spot was higher than the edge, making AgNPs more difficult to move at the middle of the spot than on the edge. As a result, the smear of Ag NPs stains became distorted at the center of the spot, where GLB density was high. The SCRE occurrence for GLB following the findings study of Zhu Q. on clozapine [13]. Since clozapine was also a hydrophobic compound, the silver colloid in Zhu’s study was also water.

#### 2.2.2. CRE Pattern of Metformin

**Figure 6:**
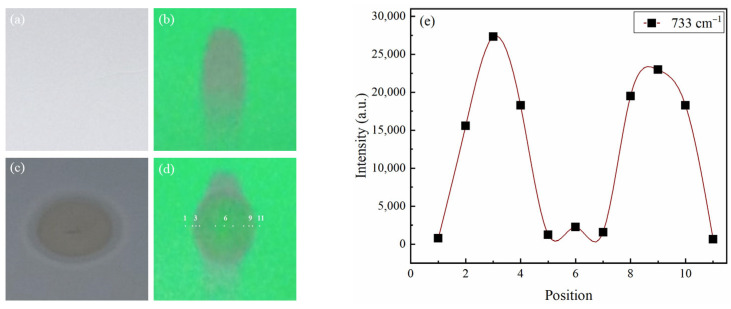
DCRE effect occurs when the silver colloid is applied at the center of the MET stain: Image of MET stain on TLC under normal light before (**a**) and after applying silver colloid (**c**); 254 nm light before (**b**) and after applying silver colloid (**d**); SERS intensity of the MET at the peak of 733 cm^−1^ at different laser positions (**e**).

The prediction for the CRE pattern of MET, as illustrated in Figure 6, was also in line with the observations on GLB. MET stain, the slightly bluish-purple spot in Figure 6b,d, clearly changed its shape after silver colloid addition. Figure 6b shows a Gaussian-like distribution of TLC spots of MET, whereas Figure 6d shows a typical coffee drop pattern. This was because MET is hydrophilic, when colloidal silver was applied to the center of the MET stain, MET particles at the center of the TLC stain had moved outside, leaving it blank at the center and aggregated on the edge of the “coffee ring”. Similarly, the AgNPs are also distributed at the edge of the coffee ring, as observed in Figure 6c. This typical double CRE was evident in Figure 6c,d, and totally in agreement with DCRE reported in our previous study on sildenafil [14].

### 2.3. CRE-Based TLC-SERS Method Optimization

The CRE-based optimization included two most important selections: (1) Selection of the position of silver colloid installation to create a better “hotspot”; and (2) Determination of the “hotspot” position with SERS signal. The highest SERS signal would be at the “hotspot” where the highest density of both analyte and AgNPs. For GLB, the silver colloid was installed in two positions: at the center (Figure 4) and on the edge of the spot (Figure 5). For the selection of “hotspot”, the SERS signal was measured at different positions numbered from 1 to 8 for GLB (Figure 4 and Figure 5) and 1 to 10 for MET (Figure 6).

#### 2.3.1. CRE-Based Optimization for Glibenclamide

As described in Section 2.2.1, since the SCRE occurred for GLB, if silver colloid was instilled from the center as in Figure 4, the distribution of GLB particles and AgNPs was in reversed direction. AgNPs accumulated at the highest density at the edge (position 5, not position 8, because of the “peanut shape” instead of the “ring shape” (Figure 4d). Meanwhile, GLB accumulated more at the center. Figure 4e presents the intensity of the Raman shift at 1593 cm^−1^ showing that higher signals were obtained at position 3 (with high GLB density) and position 5 (with higher AgNP density). But the highest SERS signal at position 5 was still weak.

Due to the reversed distribution of two types of particles, we also instilled silver colloid on the edge of the GLB spot (Figure 5). In such a case, the density of both types of particle would increase in the same direction, i.e., from 1 to 5 as in Figure 5d. The SERS intensities at 1593 cm^−1^ measured at different positions are presented in Figure 5e. The results were as expected. The highest intensity was obtained at the edge (position 5), where the density of both GLB and AgNPs was highest. When AgNPs were applied at the boundary of GLB, the spreading speed of colloidal silver on the TLC plate was faster, creating a circular “ring shape” in Figure 5b, not a “peanut shape” as in Figure 4b. This was because the density of GLB molecules at position 1 was low, so it did not prevent the colloidal silver solution from spreading rapidly. In addition, this marginal pattern allowed the colloidal silver solution to overlap the central region of the GLB stain where the GLB particle density was highest (Figure 5e).

In summary, for hydrophobic GLB, in the TLC-SERS analysis using aqueous silver colloid, the SCRE effect was evident. Accordingly, the application of silver colloids at the GLB trace edge offered the best SERS signal. At the same time, the position of the laser beam would be at the center of the GLB trace along with the boundary ring of colloidal silver.

#### 2.3.2. CRE-Based Optimization for Metformin

In contrast to GLB, for DCRE in MET analysis, the best position for silver colloid installation was the center of MET stain (similarly to sildenafil in [15]). Figure 5e presents the intensity of the highest Raman shift at 733 cm^−1^ at positions from 1 to 10 (from one edge to the other edge of the spot). Positions 3 and 9 recorded the strongest SERS intensity due to the laser position at the center of the colloidal silver ring with the highest MET molecular density (Figure 5e). Due to the high intensity of the signal, the time to record the SERS signal of the MET is only from 5 s to 20 s, which is shorter than that of the GLB.

In conclusion, the same colloidal silver solution must have a small colloidal position adjustment suitable for each substance as follows. For MET with DCRE pattern, AgNP colloidal instillation was at the center of the stain, and the laser projection position at the colloidal silver should be instilled at the edge and far from the center of the trace, and the laser location was at the colloidal silver border but near the center of the GLB spot.

### 2.4. Validation of the Detection Method by TLC-SERS

#### 2.4.1. Selectivity

For the selectivity test, a blank herbal matrix was prepared, in which all the herbal ingredients commonly found in a formula for lowering blood sugar were collected. The matrix was treated in the same sample treatment protocol for the real market sample (presented in Section 3.3), to obtain matrix extract. On the same TLC plate, the matrix extract, MET and GLB standard solution, blank matrix spiked with standard solutions, and the real sample was introduced. After TLC development, spots with respected R_f_ of MET and GLB on all traces (matrix, standards, spiked matrices) were marked. Measurements were performed without AgNP introduction, and the signal was not detected. Silver colloid was then introduced on the TLC plate on all marked spots, as well as a blank spot (blank TLC plate to measure the signal of silica gel with AgNPs). The blank spot was used for matrix subtraction.

The results showed that the herbal matrix showed no peaks according to MET and GLB typical Raman shifts, whereas the spiked matrices represented the corresponding typical Raman shifts compared to the standards.

#### 2.4.2. Limit of Detection

The standard solutions of MET and GLB were diluted and introduced on the same TLC plate. The lowest concentration of analyte that peaks corresponding to typical Raman shifts of MET and GLB could still be detected was recorded as LOD concentration. The LOD was found at 0.5 μg/spot for MET and 1 μg/spot for GLB.

### 2.5. Analysis of GLB and Adulterated MET in Herbal Products

To test the feasibility of the developed TLC-SERS method, seven real samples of herbal products were purchased from pharmacies and online. Samples were prepared as described in Section 3.3 and analyzed using the developed TLC-SERS method and examined in parallel using HPLC analysis.

#### 2.5.1. CRE-Based TLC-SERS Analysis of Real Samples

All seven products were analyzed using the developed TLC-SERS method. The TLC chromatograms of the standard mixture and seven samples were illustrated as complementary data in Table S1 and Figure S1. The separation spot of GLB was observed at the position of R_f_ = 0.77, while R_f_ = 0.05 corresponded to MET. GLB and MET were fully separated from other components in the placebo sample spiked with MET and GLB standards, as well as from all components in adulterated herbal products.

**Figure 7:**
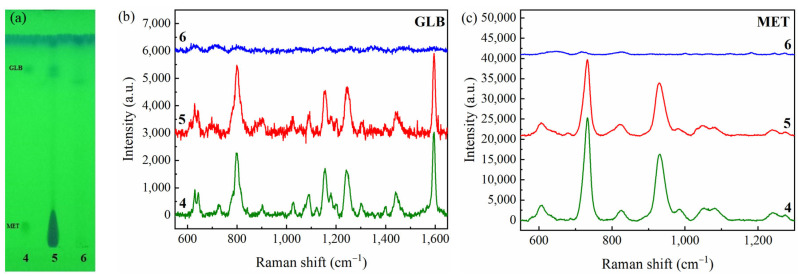
(**a**) TLC image of the 4-standard mixture, 5-positive sample (adulterated with both MET and GLB), and 6-negative sample (not adulterated); (**b**) SERS spectra of traces 4, 5, 6 at R_f_ = 0.77 for GLB; (**c**) SERS spectra of traces 4, 5, 6 at R_f_ = 0.05 for MET.

Out of seven collected samples, four samples were adulterated with anti-diabetic drugs, among which three products were adulterated with GLB, and one product was even adulterated with both compounds. This result proved the feasibility and usefulness of the simultaneous analytical method for MET and GLB. The chromatogram of the standard (trace 4), positive sample (trace 5), and negative sample (trace 6) is presented in Figure 7a. SERS spectra of GLB and MET after separation were shown in Figure 7b,c. It could be observed from the spectra that Raman peaks at 805, 1155, 1241, and 1593 cm^−1^ are assigned to the C-H bending, sulfonyl stretching, and C=O stretching deformation vibration of GLB, and the peaks at 733 and 932 cm^−1^, which are associated with the N-H wagging deformation vibration of MET. Trace 5 (adulterated with both MET and GLB) exhibited a well-resolved GLB spot but a very tailing MET spot, due to a large amount of adulterated MET in the sample. The spot tailing for the positive sample (trace 5, Figure 7a) may influence the quantitation of adulterated compounds. However, the detection of adulterated MET in real samples was not only uninfluenced by the peak tailing in this case but was even more reliable at such high concentrations. Due to the high concentration of adulterated MET, the MET spectrum of the sample (red line, Figure 7c) matched very well with the MET spectrum of the standard (green line, Figure 7c), showing the reliable confirmation of MET in the sample.

#### 2.5.2. Parallel HPLC Analysis of Real Samples

For comparative analysis, the same group of samples was analyzed in parallel using an HPLC method. Among seven tested herbal products, three samples were found positive for GLB, and one sample was positive for both MET and GLB. The analytical results were completely in line with results obtained with the TLC-SERS method (Table S1). An example is illustrated in Figure S2. The chromatogram of the real sample (No. 5 in Figure S1 and Table S1) adulterated with both compounds showed two peaks coinciding with the retention time of MET and GLB (Figure S1a), with peak purity ≥99%, and an analyte spectral superposition coefficient compared with standard ≥99% for both (b) MET and (c) GLB. Among seven samples, two samples (No. 5 and 1 in Table S1) were adulterated at high doses of GLB at 7.87 and 8.41 mg/dose, respectively, higher than the usual therapeutic dose of GLB in adults of 2.5–5.0 mg/day.

## 3. Materials and Methods

### 3.1. Chemical Reagents and Materials

Glibenclamide (GLB) (99.70%) and metformin hydrochloride (MET) (99.5%) national reference standards were purchased from the National Institute of Drugs Quality Control of Vietnam (NIDQC, Hanoi, Vietnam). Silver nitrate (AgNO_3_), sodium citrate, dimethyl formamide (DMF), and organic solvents at analytical grade and silica gel 60 F254 TLC plates were purchased from Merck (Darmstadt, Germany).

### 3.2. Apparatus and Equipment

Raman spectra were recorded with a LabRAM HR μ-Raman (Horiba Jobin Yvon, Longjumeau, France) with a 633 nm laser source. HPLC analyses were performed on a Shimadzu LC 20AD HPLC system (Shimadzu, Kyoto, Japan) equipped with PDA detector SPD-M20A using an InertSustainTM column C18 (250 mm × 4.6 mm, 5 μm) from GL Sciences (Tokyo, Japan). Other equipment included a Sartorius TE 214S analytical balance (d = 0.1 mg) (Sartorius, Goettingen, Germany); a Labinco BV L46 vortex shaker (Labinco, Breda, The Netherlands); an Elma D-78224 Singen (Elma, Singen, Germany); and a Kubota 6500 centrifuge (Kutoba, Osaka, Japan).

### 3.3. Sample Preparation

Seven herbal products for supportive diabetes treatment were purchased at pharmacies or via the Internet. Sample preparation was conducted as follows: the sample was finely ground and homogenized. An amount of 0.5 g of sample was weighed into a centrifuge tube, where 25 mL of methanol was added, vortexed for 5 min, sonicated for 20 min at room temperature, centrifuged for 5 min at 13,522× *g*, and filtered through a 0.45 m membrane before analysis.

### 3.4. Silver Colloid Preparation

Colloidal silver suspension in water was prepared according to previously published research [15]: 94 mL of distilled water was heated to 100 °C in a reflux condenser with vigorous magnetic stirring. After boiling, 2 mL of 0.1 M silver nitrate solution was dropwise added, followed by 4 mL of 1% sodium citrate solution. Reflux heating was maintained for 40 min, then naturally cooled down to room temperature to obtain nano-silver colloid. The silver colloid was stored in the refrigerator and used within 6 months with acquired stability for TLC-SERS measurement.

### 3.5. Analytical Conditions

#### 3.5.1. For TLC-SERS Method

Silica gel 60 F254 with a size 20 × 10 cm TLC plate was used as the stationary phase, and a mixture of n-butyl acetate/MeOH/formic acid (11:2.5:1.5, *v*/*v*/*v*) as the mobile phase, with 5 µL sample volume; TLC spots were detected under a 254 nm UV lamp. The TLC spot equivalent to the retardation factor (Rf) of glibenclamide and metformin was marked under UV illumination at 254 nm in UV Cabinet 4 (Camag, Muttenz, Switzerland), then 1.5 µL of silver colloid was applied directly on the marked spot on the TLC plate. Finally, the SERS spectrum for each separated spot was recorded using LabRAM µ-Raman with a CCD camera and an input laser beam of 633 nm. The obtained data were processed and interpreted by LabSpec 5 and Origin software. Validation of the selectivity and limit of detection was conducted according to AOAC.

#### 3.5.2. For the HPLC-DAD Method

Experimental conditions were as follows: column chamber temperature: 40 °C; sample injection volume: 20 µL; flow rate: 1 mL/min; analysis time: 25 min. The mobile phase solvent system consisted of methanol (A) and sodium dihydro phosphate buffer pH 3.0(B) at a ratio of 70:30 by volume.

## 4. Discussion

### 4.1. Remarks from TLC-SERS Method Development

For the TLC method in Section 2.1.1, since MET and GLB are very different in hydrophobicity, the challenge was not the separation of the two compounds. On the contrary, choosing a mobile phase that can elute both compounds on the same TLC plate was an issue to consider. The main purpose of the TLC separation, as explained in the introduction, was to eliminate the influence of the fluorescent active herbal components. Therefore, it was important to separate both compounds from as many herbal compounds as possible. To do that, the herbal placebo matrix sample was prepared, which included the herbal ingredients most commonly found in herbal products on the market. With the chosen TLC mobile phase, most of the ingredients either stay at the starting point on the TLC plate or elute very fast with the mobile phase. The influence of their fluorescence was minimized. Therefore, although the retention factor of MET (0.05) was not a recommended value for TLC analysis, the chosen mobile phase was still accepted.

Another interesting point was noticed during the TLC method development, which was the sample preparation for traditional charcoal-coated pills. It was previously common in the production of traditional pills to include charcoal in the coating of the pills. This charcoal has very high fluorescence properties, and although it remained on the starting point of the TLC plate, well separated from both compounds, its fluorescence may have been sufficiently high to mask the SERS signals. Therefore, especially for this type of product, it is necessary to add a washing step with water. The pills were put into a test tube containing water, shaken by hand for 30 s, and then taken out of the water, freshly dried before grinding. With this easy step, the charcoal coating was removed, and the sample treatment could be carried out as described in Section 3.3.

For the selection of typical Raman shifts for identification, it is known that peaks of typical Raman shifts in SERS spectra can slightly differ from those of the original Raman spectra. Therefore, it is necessary to compare SERS and Raman spectra of the reference standards to select typical Raman shifts for the identification of each compound. In Section 2.1.3, results showed that the vibrational frequencies in SERS spectra for both GLB and MET were in good compliance with Raman spectra. The typical shifts chosen for identification were reliable, as presented in Table 1.

### 4.2. Remarks from CRE-Based TLC-SERS Method Optimization

In this study, the predictions of CRE patterns for GLB and MET were proven practically. It was obvious that the selection of the position for silver colloid droplet introduction (at the center or on the edge of the TLC spot) and the selection of the position for Raman measurement (at the center or on the edge of the coffee ring) significantly influence the sensitivity of the TLC-SERS method.

It was well known that the signal on the edge of the coffee ring can be much higher than in the center; therefore, if the CRE is disregarded, the sensitivity of the analytical method would be significantly decreased. In this study, it was shown that the difference in intensities between the edge and the center can be almost 3000 times in DCRE (Figure 6e, position 1 versus 5) instead of only 80–100 times in the SCRE (Figure 4e, position 1 versus 5).

It can also be confirmed that the position to introduce silver colloids should be chosen appropriately according to the CRE pattern. Comparing the intensity obtained at the edge position of the AgNP ring (position 5) in Figure 4 and Figure 5 with the same concentration of GLB, it was shown that the intensity was 5 times higher in Figure 5 than in Figure 4. This means if the CRE pattern is correctly determined and the dropping position of the silver colloid is adjusted, the sensitivity can be increased by 5 times easily.

Although the occurrence of the CRE pattern based on the hydrophobicity of the analytes observed in this study was quite explainable and even predictable for SERS applications, the importance of the determination of the CRE pattern in TLC-SERS applications towards the sensitivity of the method was not clearly explained in previous TLC-SERS studies. Especially for TLC practitioners, the adsorption of the analytes on silica is believed to prevent analytes from moving easily on TLC plates. Therefore, the de-adsorption of analytes in silver colloid solution is not an obvious phenomenon. In other words, in a TLC-SERS analysis, we cannot be sure how hydrophobic a compound must be to ensure that the SCRE or DCRE occurs. GLB and MET in this study can be used again as a typical example of the SCRE and DCRE to remind the TLC-SERS users to include the CRE pattern determination step in the method development because the CRE-based optimization may significantly improve the sensitivity of the method.

Because silver colloids can be prepared in solvents other than water, more studies can be performed in the future to consolidate the CRE pattern prediction to facilitate TLC-SERS users in their method development process.

**Figure 8:**
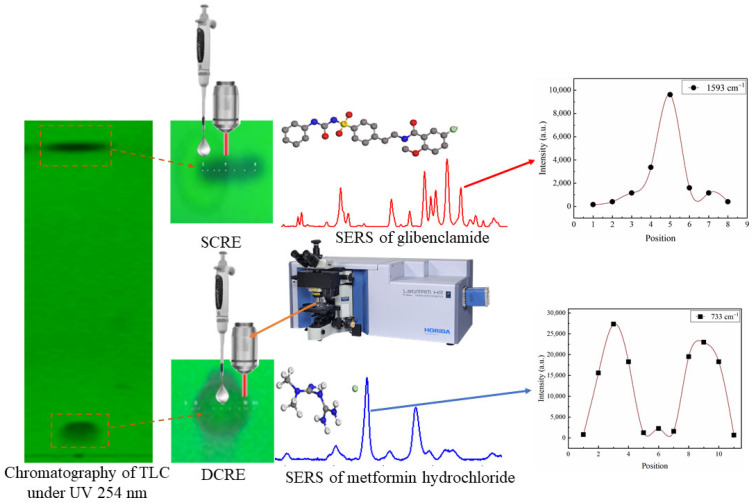
CRE-based TLC-SERS analysis of GLB and MET.

In conclusion, the CRE-based optimization of TLC-SERS is summarized as shown in Figure 8. It comprises two factors: (1) the position of the silver colloid droplet: at the edge of the TLC spot for SCRE and the center of the TLC spot for DCRE; (2) the position of the light beam for SERS measurement: at the center of the TLC spot for both the SCRE and DCRE, which is also the edge of the silver colloids ring for both the SCRE and DCRE. As illustrated in the study, SCRE was observed for a hydrophobic compound, GLB, while DCRE was observed for a hydrophilic compound, MET.

### 4.3. Remarks from TLC-SERS Method Application in Real Samples

The ratio of adulterated herbal products detected was still very high (four adulterated out of seven tested samples), although the adulteration issue for diabetic drugs is not new in Vietnam. Both MET and GLB have been included in the official list of compounds prohibited for use in dietary supplements. The reason for a high ratio of adulteration may relate to the method of collecting samples. Products with official “Certificates of Food Hygiene and Safety” issued by the National Department of Food Safety were not included. All products were purchased via the Internet and available only through online sales.

This fact confirmed again the importance of proposing rapid methods for the detection of adulteration, especially for products available through online marketing. On the other hand, the presence of both MET and GLB in one product has proved the importance of the simultaneous detection of these compounds in one analytical method.

## 5. Conclusions

In the current study, the CRE patterns were compared in two anti-diabetic drugs with differences in hydrophobicity, i.e., glibenclamide and metformin. Using aqueous silver colloid as an enhancement tool for TLC-SERS analysis, the occurrence of the SCRE for hydrophobic GLB, in contrast to DCRE for more hydrophilic MET, was observed. The CRE demonstrated that colloidal silver solution formed more hotspots, so the established method exhibited excellent TLC-SERS performance. The colloidal silver solution could be used to detect both hydrophilic and hydrophobic active substances but must be adjusted accordingly in terms of colloidal technique and laser position shine on the stain. For the SCRE with GLB, the silver colloid installation should be applied on the edge of the TLC analyte spot, whereas for DCRE with MET, the application should be at the right center of the TLC plot for best SERS performance. These findings can be useful for the CRE-based TLC-SERS method development for other drug compounds in the future.

This method was successfully applied to the analysis of seven real samples collected from the market. Four herbal products were found to be adulterated with GLB or MET, among which one sample was adulterated with both MET and GLB at the same time. The results were in line with the results of parallel HPLC analysis for comparison.

## Figures and Tables

**Table 1:** Vibration frequencies obtained from direct Raman spectra of MET and GLB in powder and SERS spectra of MET and GLB on TLC spots (with silver colloidal enhancement) compared to reference ^a^ [20]; ^b^ [21]; ^c^ [22]; ^d^ [23].

Samples	Powder Raman Shift (cm^−1^)	Solution SERS Shift (cm^−1^)	Ref.	Description
GLB	644	643	653 ^a^; 653 ^b^	C-Cl stretching
	745	726	763 ^a^; 787 ^b^	C-H bending
	805	802	816 ^a^; 810 ^b^	C-H bending
	839	839	842 ^a^; 843 ^b^	C-H bending
	903	903	855 ^a^; 878 ^b^	C-H bending
	1014	1008	1027 ^a^; 1030 ^b^	C-H bending
	1054	1025	1053 ^a^; 1088 ^b^	C-O stretching
	1090	1087	1110 ^a^	C-H bending
	1151	1155	1163 ^a^;1158 ^b^	Sulfonyl stretching
	1244	1241	1261 ^a^; 1203 ^b^	C-H bending
	1362	1368	1344 ^a^; 1336 ^b^	C-H bending
	1394	1398	1399 ^a^;	Sulfonyl stretching
	1435	1441	1433 ^a^; 1442 ^b^	C=C stretching
	1523	1545	1530 ^b^	C=C stretching
	1589	1593	1592 ^a^; 1582 ^b^	C=O stretching
MET	418	409	561 ^a^, 490 ^c^; 428 ^d^	C-N-C deformation
		606	622 ^a^, 623 ^d^	C-N-C deformation
	733	733	739 ^a^, 750 ^c^	N-H wagging
		826	849 ^a^,	N-H wagging
	931	932	950 ^c^; 931 ^d^	N-H wagging
		987	1003 ^a^	N-H wagging
		1047	1033 ^a^; 1057 ^d^	C-N stretching
		1081	1045 ^d^	C-N stretching
		1240	1209 ^a^; 1248 ^d^	C-N stretching
		1275	1300 ^c^; 1266 ^d^	C-N stretching

## Data Availability

No privacy or ethical restriction is required for this research data.

## Supplementary Materials

The following supporting information can be downloaded at: https://www.mdpi.com/article/10.3390/molecules28145492/s1, Figure S1: Chromatography of mixture of MET and GLB standard (4) and seven samples (1-3, 5-8) with two samples adulterated with GLB (7 and 8) and one sample adulterated with both GLB and MET (5); Figure S2: (a). chromatography of real samples adulterated with both MET and GLB; (b). purity and ratio of UV spectra between MET peak at Rt = 2.6 min; (c) purity and the ratio of UV spectra between GLB peak at Rt = 12.1 min; Table S1. TLC-SERS analytical results of real samples.
