# Supplementary material for: Single versus Double Coffee-Ring Effect Patterns in Thin-Layer Chromatography Coupled with Surface-Enhanced Raman Spectroscopic Analysis of Anti-Diabetic Drugs Adulterated in Herbal Products
Source: Molecules. 2023 Jul 18;28(14):5492. doi: 10.3390/molecules28145492 (PMC10386024; doi:10.3390/molecules28145492)
Supplement: Supplementary file 1 [file molecules-28-05492-s001.zip › molecules-2467581-supplementary.pdf]

Table S1. TLC-SERS analytical results of real samples

**TLC-SERS method**		**HPLC method**		
**Samples**	**Analytical substances**	**Analytical substances**	**Content (mg/g)**	**Content (mg/dose)**
1	GLB	GLB	4.54	7.87
2	-	-	-	-
3	-	-	-	-
5	GLB	GLB	1.09	8.41
	MET	MET	117.74	470.59
6	-	-	-	-
7	GLB	GLB	0.27	0.28
8	GLB	GLB	0.25	0.26

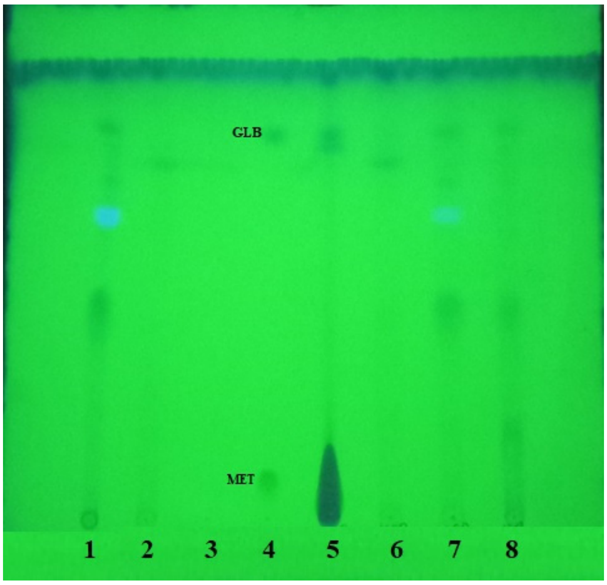

Figure S1. Chromatography of a mixture of MET and GLB standard (4) and 7 samples (1-3, 5-8) with 2 samples adulterated with GLB (7 and 8) and one sample adulterated with both GLB and MET (5)

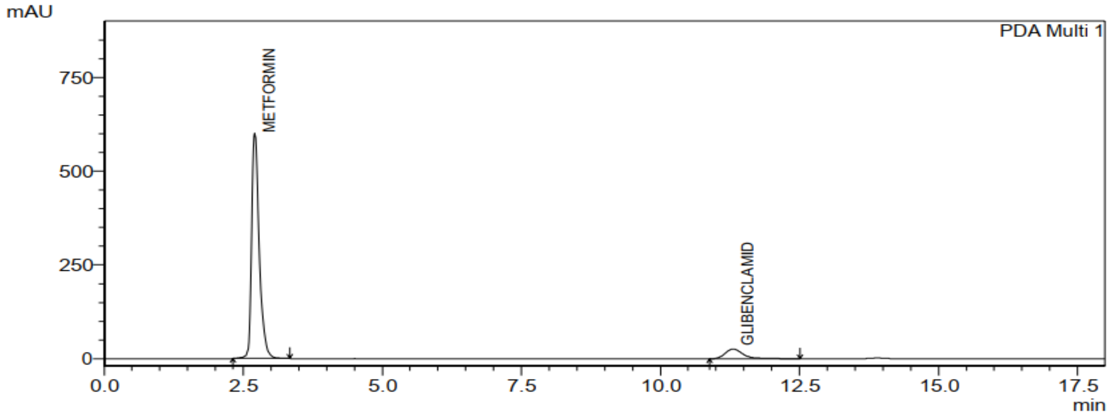

(a)

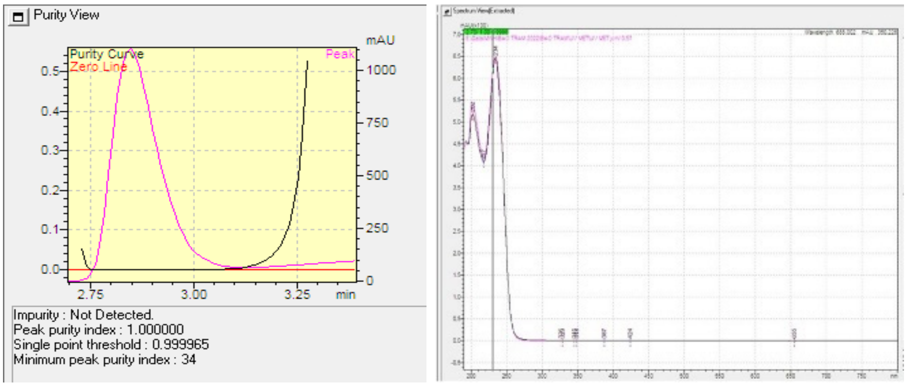

(b)

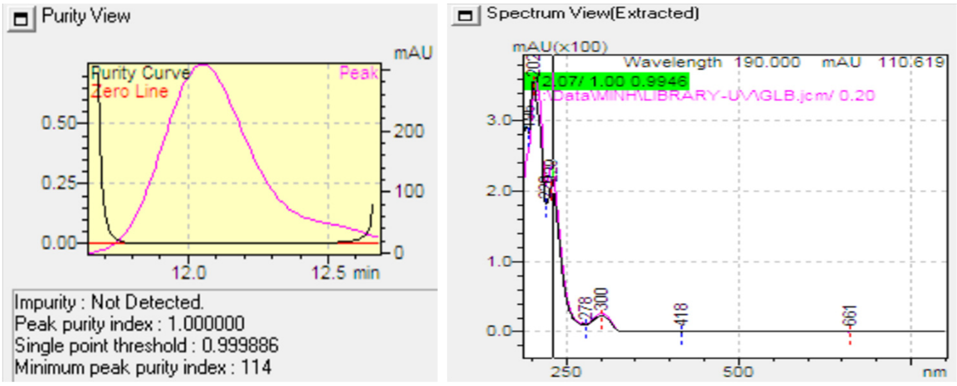

(c)

Figure S2.(a). chromatography of real sample adulterated with both MET and GLB; (b). Purity and ratio of UV spectra between MET peak at Rt= 2.6 min; (c) Purity and the ratio of UV spectra between GLB peak at Rt= 12.1 min.
